# A Narrative Review of the Management of Type 2 Myocardial Infarction and Myocardial Injury in Medical Patients in the UK

**DOI:** 10.7759/cureus.100853

**Published:** 2026-01-05

**Authors:** Muhammad Usman, May Lai Phyo, Anisha Srirangan, Mostofa Kowshik, Casserene E Shen Yeow, Pooja Prashant Kirtani

**Affiliations:** 1 Acute Medicine, Norfolk and Norwich University Hospital, Norwich, GBR; 2 General Medicine/Cardiology, Norfolk and Norwich University Hospital, Norwich, GBR

**Keywords:** acute illness, demand ischemia, diagnosis and management, long-term prognosis, type 2 myocardial infarction

## Abstract

Type 2 myocardial infarction (T2MI) is defined as myocardial injury occurring due to an imbalance between myocardial oxygen supply and demand rather than acute atherothrombotic plaque rupture. It is frequently encountered in acutely unwell medical patients and is associated with adverse short- and long-term outcomes, including increased inpatient, 30-day, and one-year mortality. Diagnosis and management remain challenging due to heterogeneous clinical presentations, frequent coexistence with systemic illness, and overlap with acute non-ischaemic myocardial injury. In the UK, there is currently no national guidance specific to T2MI, resulting in variability in diagnostic approaches and management strategies, with clinicians often extrapolating from acute coronary syndrome pathways and international consensus statements. This narrative review synthesises contemporary evidence from observational studies, guideline documents, and expert consensus to discuss the pathophysiology, diagnosis, investigation, and management of T2MI in medical inpatients, highlighting key uncertainties and areas requiring future research.

## Introduction and background

The Fourth Universal Definition of Myocardial Infarction (2018) defines type 2 myocardial infarction (T2MI) as an acute myocardial injury accompanied by clinical evidence of myocardial ischaemia, resulting from myocardial oxygen supply-demand mismatch rather than acute coronary thrombosis [1]. Common precipitating factors include sepsis, hypoxia, anaemia, hypotension, and tachyarrhythmias. In contrast, acute myocardial injury refers to a rise and/or fall in cardiac troponin concentrations above the 99th percentile upper reference limit without accompanying clinical evidence of ischaemia [1].

T2MI is increasingly recognised in acutely unwell medical patients and is considered frequent in this population, particularly in older adults with multiple comorbidities. The condition carries an adverse prognosis, with observational cohorts and meta-analyses consistently demonstrating higher short- and long-term mortality compared with type 1 myocardial infarction (T1MI) [2]. This excess mortality is thought to reflect a combination of advanced comorbidity burden, severity of the precipitating illness, and variability in management strategies rather than myocardial injury alone.

Reported prevalence of T2MI varies widely, ranging from approximately 2% to over 50% in different studies [3]. This variation is largely attributable to differences in diagnostic definitions, patient populations studied, and methods of adjudicating myocardial injury. Misclassification remains common, with a substantial proportion of cases labelled as T2MI later reclassified as acute myocardial injury when diagnostic criteria are applied rigorously [4]. Such misclassification has important implications for treatment decisions, research comparability, and outcome assessment.

In the UK, there is no dedicated national guidance addressing the diagnosis and management of T2MI. As a result, clinicians commonly rely on triangulation of National Institute for Health and Care Excellence (NICE) acute coronary syndrome guidance [5], European Society of Cardiology (ESC) recommendations [6], and American Heart Association (AHA) scientific statements [3]. This narrative review aims to synthesise contemporary evidence to clarify diagnostic principles, investigative strategies, and pragmatic management considerations for T2MI in UK medical patients, while highlighting key evidence gaps that contribute to ongoing clinical uncertainty.

## Review

Search strategy and methods

This narrative review was conducted using a structured but non-systematic approach. The literature was identified through searches of PubMed, MEDLINE, and relevant guideline repositories, focusing on studies published from 2017 onwards to reflect contemporary practice in the era of high-sensitivity troponin assays. Search terms included combinations of "Type 2 myocardial infarction", "myocardial injury", "supply-demand mismatch", and "troponin". Key consensus statements, observational cohort studies, meta-analyses, and guideline documents were prioritised. Articles were selected based on relevance to the diagnosis, investigation, and management of T2MI, with particular attention to applicability within UK medical inpatient settings.

Pathophysiology

T2MI arises from myocardial oxygen supply-demand mismatch in the absence of acute atherothrombotic coronary occlusion. Systemic stressors may increase myocardial oxygen demand, such as tachyarrhythmias or sepsis, or reduce oxygen supply through hypotension, hypoxaemia, or anaemia. Unlike T1MI, where plaque rupture and thrombosis result in abrupt coronary obstruction, T2MI reflects functional ischaemia rather than primary structural coronary pathology.

However, underlying coronary artery disease (CAD) is common in patients with T2MI and plays a significant contributory role by reducing coronary flow reserve, thereby lowering the threshold for ischaemia during systemic stress [1]. Autonomic dysregulation and coronary microvascular dysfunction may further impair myocardial perfusion, particularly in older patients and those with diabetes or chronic inflammatory states.

At the cellular level, oxygen deprivation disrupts oxidative phosphorylation, leading to ATP depletion, impaired ionic homeostasis, and activation of cell injury pathways [7]. Prolonged or severe mismatch may progress to myocyte necrosis, resulting in troponin release into the circulation. Although troponin elevations in T2MI are often lower than in T1MI, there is substantial overlap in magnitude and kinetics, limiting their utility in distinguishing between MI subtypes [8]. Systemic inflammation may further modulate myocardial oxygen utilisation, amplifying injury in critically ill patients.

Risk factors and precipitating factors

Baseline risk factors for T2MI overlap substantially with those for T1MI, including advanced age, hypertension, diabetes, dyslipidaemia, chronic kidney disease, and established CAD [9]. Among these, prior T1MI is one of the strongest predictors of subsequent T2MI, as demonstrated in observational cohort studies [9].

Acute precipitating factors should be distinguished from baseline risk factors and include sepsis, anaemia, hypoxia, hypotension, acute heart failure, and tachy- or brady-arrhythmias [8,9]. Many of these precipitants are potentially modifiable, and their prompt identification and management represent the cornerstone of treatment. In contrast, non-modifiable factors such as age and pre-existing cardiovascular disease primarily inform risk stratification and prognosis. Available evidence suggests that the risk profile of T2MI in UK medical inpatients mirrors that observed in other high-income healthcare settings, though direct comparative data remain limited.

Clinical presentation

Clinical presentation of T2MI is heterogeneous and often dominated by symptoms of the underlying precipitating illness. Classical anginal chest pain is less frequent than in T1MI, and many patients present with atypical features such as dyspnoea, delirium, syncope, or isolated troponin elevation [3,10,11]. Silent or minimally symptomatic presentations are common, particularly in older adults and those with diabetes, contributing to delayed diagnosis.

Distinguishing symptoms attributable to myocardial ischaemia from those related to systemic illness remains challenging. Currently available risk stratification tools have limited validation in T2MI populations, and their ability to guide management decisions remains uncertain. This diagnostic ambiguity contributes to variability in clinical practice and highlights an important evidence gap.

Investigations

Electrocardiography (ECG) remains a first-line investigation. Non-specific ST-segment or T-wave changes, atrial fibrillation, and tachyarrhythmias are commonly observed in T2MI, though ECG findings often reflect the underlying systemic condition rather than focal coronary occlusion [3].

Cardiac troponin assays confirm acute myocardial injury through demonstration of a rise and/or fall in troponin levels. Serial testing is valuable in confirming acuity but does not reliably distinguish T1MI from T2MI due to substantial overlap in absolute values and delta changes [3,11-13]. Troponin kinetics, therefore, have limited clinical utility beyond confirming acute injury.

Echocardiography is underutilised but can provide valuable information, particularly when performed early. Regional wall motion abnormalities may support ischaemia, while identification of structural heart disease, such as aortic stenosis or cardiomyopathy, can clarify mechanisms contributing to supply-demand mismatch [3,12,13].

Myocardial perfusion imaging may be considered selectively in patients with diagnostic uncertainty where results would alter management. Its use must be balanced against cost, contrast exposure, and renal risk [3,12,13].

Coronary angiography offers a definitive assessment of coronary anatomy and can identify plaque rupture, embolism, vasospasm, or stable CAD. However, routine angiography for all suspected T2MI has not been shown to improve outcomes, and decisions should be individualised based on clinical context, comorbidity burden, and likelihood of benefit [12,13].

Diagnosis

Diagnosis of T2MI requires a rise and/or fall in cardiac troponin with at least one value above the 99th percentile, accompanied by evidence of acute myocardial ischaemia and a mechanism consistent with supply-demand mismatch rather than acute coronary thrombosis [1]. Evidence of ischaemia may include ischaemic symptoms, new ECG changes, pathological Q waves, or imaging evidence of new regional wall motion abnormalities.

Common diagnostic pitfalls include misattributing troponin elevation to chronic myocardial injury or systemic illness alone and failing to document objective evidence of ischaemia. Coexisting CAD increases diagnostic uncertainty and often lowers thresholds for further investigation.

Management

There is currently no UK-specific, evidence-based guideline for the management of T2MI. NICE guidance focuses on acute coronary syndromes [5], while clinicians commonly extrapolate from ESC and AHA documents [3,6]. This has resulted in heterogeneity in practice and uncertainty regarding optimal management strategies.

The primary management principle in T2MI is the identification and treatment of the precipitating cause of myocardial oxygen supply-demand mismatch. This includes prompt treatment of sepsis, correction of anaemia or hypoxia, rate or rhythm control of arrhythmias, and optimisation of haemodynamics.

Key management differences from T1MI include uncertainty regarding the routine use of dual antiplatelet therapy, anticoagulation, and invasive coronary intervention. Overtreatment may expose patients to bleeding risk, drug interactions, and procedural complications without clear benefit, particularly in older or multi-morbid populations [3].

Coronary angiography may benefit selected patients, particularly those with recurrent ischaemia, haemodynamic instability, or high suspicion of underlying obstructive CAD. However, evidence supporting routine invasive strategies in T2MI is lacking, and patient selection remains a priority for future research.

Secondary prevention therapies, such as statins, beta-blockers, and antiplatelet agents, have uncertain benefit in T2MI, and their use should be individualised. Priority areas for future research include prospective trials evaluating invasive strategies, secondary prevention, and structured diagnostic pathways.

## Conclusions

T2MI is a clinically important and increasingly recognised entity in acutely unwell medical patients, characterised by myocardial oxygen supply-demand mismatch and associated with adverse outcomes. Diagnostic and management challenges are compounded by heterogeneous presentations, frequent comorbidities, and a lack of UK-specific guidance.

Current practice relies on individualised clinical judgement and extrapolation from existing acute coronary syndrome frameworks. Management should prioritise treatment of the underlying precipitating illness while maintaining vigilance for coexisting CAD. Future research should focus on refining diagnostic criteria, identifying patient subgroups most likely to benefit from invasive assessment or secondary prevention, and developing evidence-based UK guidance to improve consistency and outcomes.
